# Work-Related Stress and Glucose Regulation in Air Traffic Control Officers: Implications for Medical Certification

**DOI:** 10.3390/biomedicines13092125

**Published:** 2025-08-30

**Authors:** Paola Verde, Laura Piccardi, Sandro Gentile, Graham A. Roberts, Andrea Mambro, Sofia Pepe, Felice Strollo

**Affiliations:** 1 Aerospace Medicine Department, Aerospace Test Division, Mario De Bernardi AFB, Pratica di Mare, 00071 Rome, Italy; 2 Department of Psychology, Sapienza University of Rome, 00185 Rome, Italy; sofia.pepe@uniroma1.it; 3 IRCCS San Raffaele Cassino, 03043 Cassino, Italy; 4 Precision Medicine Unit, Vanvitelli University, 81100 Naples, Italy; s.gentile1949@gmail.com; 5 Nefrocenter Research Network, 80131 Torre del Greco, Italy; 6 Irish Aviation Authority, Aeromedical Section, D02 T449 Dublin, Ireland; graham.roberts@hse.ie; 7 HRB-Clinical Research Facility, University College Cork, T12 K8AF Cork, Ireland; 8 Diabetes Research Unit, Grove Building, Swansea University, Swansea SA2 8PP, UK; 9 Anaesthesiology Department, San Camillo Forlanini Hospital, 00152 Rome, Italy; andreamambro@scamilloforlanini.rm.it; 10 Endocrinology Department, IRCCS San Raffaele Pisana, 00163 Rome, Italy; felix.strollo@gmail.com

**Keywords:** air traffic control operator, stress, insulin, diabetes mellitus, continuous glucose monitoring

## Abstract

**Background/Objectives:** Following the recent publication of reassuring outcomes from the ARA MED 330 protocol regarding long-term insulin use in pilots, combined with continuous advancements in diabetes technology, European aeromedical examiners are increasingly optimistic about establishing more flexible medical requirements for insulin-treated aviation professionals. These professionals have historically been considered unfit for duty due to hypoglycemic risks. According to current research, hypoglycemia, the primary incapacitation risk for flight crew, is considered virtually non-existent among air traffic controllers (ATCOs). Additionally, stress-induced hyperglycemia also represents a low-frequency risk in these professionals, who are experienced in managing highly stressful operational environments. This study presents a narrative review examining stress and its metabolic effects in healthy individuals, ATCOs, and people with diabetes (PwD). **Methods:** This narrative review was conducted based on a comprehensive PubMed search performed by two independent reviewers (GAR and AM) spanning January 2023 to January 2025. The search strategy focused on English-language, peer-reviewed studies involving human participants and addressed stress, glucose regulation, and occupational factors in ATCOs and people with diabetes. Additional relevant articles were identified through reference screening. A total of 33 studies met the inclusion criteria. Studies focusing solely on oxidative or molecular mechanisms were excluded from the analysis. **Results:** Stressful events consistently triggered the expected hyperglycemic reaction in both healthy individuals and PwD. However, the literature indicates ATCOs demonstrate remarkable stress resilience and adaptation to the demanding conditions of their work environment, suggesting a unique occupational profile regarding metabolic stress responses. **Conclusions:** These findings contribute valuable insights to ongoing discussions regarding aeromedical fitness standards. The evidence suggests that ATCOs may not face the same metabolic risks as flight crews, indicating that current medical certification processes for insulin-treated aviation professionals warrant reconsideration in light of this emerging evidence. This research supports the potential for more individualized, occupation-specific aeromedical standards that better reflect the actual risk profiles of different aviation roles.

## 1. Introduction

Diabetes mellitus is a metabolic disorder characterized by chronic hyperglycaemia, occurring due to defects in insulin secretion, insulin action, or both. Indeed, the condition encompasses type 1 (insulin deficiency) and type 2 (insulin resistance and/or deficiency), both resulting in persistent high blood glucose levels (hyperglycaemia). Hyperglycaemia and hypoglycaemia are central to its diagnosis, management, and complications. Chronic hyperglycaemia in diabetes leads to damage to blood vessels and organs, increasing the risk of microvascular and macrovascular complications such as retinopathy, nephropathy, neuropathy, and cardiovascular disease. Management focuses on maintaining blood glucose within a target range to prevent such complications [1,2]. Hypoglycaemia is defined as blood glucose levels below the normal range and is a frequent adverse effect of diabetes treatment, especially with insulin or sulphonylureas. Severe episodes can cause confusion, seizures, loss of consciousness, and may lead to cardiovascular events and cognitive impairment [3,4,5]. It is more common in diabetes mellitus type 1, but it can also occur in insulin-treated type 2 diabetes [3]. Both hypoglycaemia and hyperglycaemia impair cognitive function by affecting attention, executive function, and working memory—domains crucial for aviation safety [6,7,8].

While diabetes does not automatically prevent employment, safety-sensitive roles such as aviation may have restrictions for insulin-treated individuals due to their intrinsic hypoglycaemic risk. However, present management protocols and individualized assessment strategies have allowed increasingly more people with diabetes to work in these fields, provided they meet specific medical criteria and monitoring protocols [9].

Currently, ATCOs must meet the same medical requirements as other aviation professionals for certification. Following the reassuring results of the ARA MED 330 protocol for insulin-treated pilots [10], and given continuous advances in diabetes technology, updated evidence-based medical requirements are expected in Europe for insulin-treated aviation professionals previously deemed unfit for duties due to assumed hypoglycaemia-related incapacitation risk, classified as level 1 (3.0–3.8 mmol/L or 54–69 mg/dL) or level 2 (<3.0 mmol/L or <54 mg/dL) [11].

The actual hypoglycemic incapacitation risk has decreased substantially over two decades with pen devices containing fast-acting or basal insulin analogs that closely mimic pancreatic secretion, and semi-automated insulin pumps. These technologies enable patients to maintain good metabolic control within “time in range” standards (TIR) [12].

Given ATCOs’ specific working conditions, the most relevant risk may be hyperglycaemia from stress rather than hypoglycaemia. Notably, hyperglycaemia is not considered operationally threatening unless exceeding 15.0 mmol/L (270 mg/dL), the upper threshold deemed acceptable for pilot work efficiency in the ARA MED 330 protocol [10].

ATCOs face demanding schedules and a high-stress environment, which disrupts natural cortisol rhythms, crucial for stress regulation and metabolic balance. Studies show ATCOs secrete more cortisol than non-shift workers, with night shifts causing greater circadian disruption and representing additional health risks for those with diabetes [13,14,15,16]. Few studies have investigated real-life glucose management in active ATCOs with diabetes due to their systematic exclusion from operational duties. However, a recent case study reported optimal metabolic control in two insulin-treated ATCOs, demonstrating stable glucose profiles with no hypoglycaemic events and time in range values exceeding 90% during working hours [17]. These cases suggest minimal impact of operational stress on glucose variability and support re-evaluating fitness-for-duty criteria. The core argument is that modern diabetes technology has rendered hypoglycemia-related incapacitation risk negligible, warranting the re-evaluation of standards. A blanket disqualification of insulin-treated ATCOs appears anachronistic and inequitable. A rigorous, case-by-case aeromedical evaluation process, similar to Federal Aviation Administration (FAA) protocols for pilots, could safely permit qualified individuals to continue in their highly specialized roles, preserving their extensive training and expertise [17].

To explore this hypothesis further, we performed a narrative review of existing literature on stress-related metabolic effects, focusing on healthy individuals, PwD, and ATCOs’ work-related stress exposure. We selected a narrative review format to stimulate debate on this unexplored subject, concentrating on workers who developed diabetes after hiring. Given advances in diabetes treatment, these individuals should continue contributing in their roles rather than being sidelined [17].

## 2. Materials and Methods

This narrative review was conducted through a structured literature search performed independently by two reviewers (GAR and AM) between January 2023 and January 2025. The review followed SANRA criteria, a validated scale for assessing narrative review quality [18].

A structured search was conducted on MEDLINE via PubMed (https://pubmed.ncbi.nlm.nih.gov/) to identify relevant studies published in English with the following Boolean strings: “ATCO” AND “stress” (*n* = 7); “ATCO” AND “fatigue” (*n* = 5); “work” AND “stress” AND “glucose” AND “human” AND “adult” NOT “oxidative” (*n* = 48); “work” AND “stress” AND “confinement” AND “human” AND “adult” NOT “oxidative” (*n* = 3); “work” AND “stress” AND “continuous glucose monitoring” NOT “oxidative” (*n* = 4). Studies addressing oxidative stress or molecular-level mechanisms were excluded, as they were not directly relevant to the clinical and operational dimensions explored in this review. Following the retrieval of 67 titles, GAR and AM independently selected and analyzed all papers relevant to the research question. The reviewers also examined reference lists of the selected articles to identify additional relevant studies. Regular video-conference meetings were held to monitor progress, discuss potentially eligible studies that provided objective results, and reach a consensus on the final decision when disagreement occurred. PubMed was selected as the primary database due to its comprehensive indexing of clinical and physiological literature relevant to this topic. Search terms encompassed ATCOs, stress, fatigue, glucose regulation, confinement, and continuous glucose monitoring. A total of 33 documents were ultimately included in the narrative review (identified in bold in the reference list). These comprised peer-reviewed studies conducted on human participants and published primarily in English, with two exceptions: one in Portuguese and one in Spanish articles were included due to their relevance to the ATCO stress debate. Table 1 presents selected articles from the narrative review that focus on ATCOs’ workload, stress management, and physiological parameters. The selected literature specifically examined the effects of work-related stress on physiological or metabolic parameters, the impact of occupational stress in ATCOs, and the relationship between diabetes and stress in both ATCOs and the general population.

## 3. Results and Discussion

### 3.1. Stress as the Leading Aspect of Air Traffic Control Operators’ Activities

We define stress as human reactions to emotional, cognitive, and physical challenges, particularly when individuals remain vigilant against imminent dangers. The stress response is characterized by the “fight-freeze-flight” response typical of autonomic nervous system activation [32].

ATCOs operate in high-stress environments, classified by the U.S. Department of Labor at the upper fourth level [33], resulting in the vast majority of controllers (i.e., 83.6%) experiencing significant occupational stress and burnout [34], ending their careers before retirement age with stress-related work disabilities [35].

Unlike pilots and cabin crew, ATCOs do not experience pressure differences or vibrations that can contribute to incapacitation risk. However, stress from communication/management demands and environmental factors remains an integral part of their job.

Analyzing the effects observed in other office workers exposed to highly stressful job conditions, we encountered difficulty in relying on specific psychometric tests due to progress in occupational medicine that occasionally renders widely accepted tests obsolete. For example, recent studies comparing the SART (Sustained Attention to Response Task) to traditional vigilance formats have challenged current diagnosis assumptions, warranting further validation studies [36].

ATCO tasks require complex attention and high precision, influencing emotions, cognitive workload, and performance. Studies using objective measures (pupil dilation) and subjective assessments (questionnaires) have shown that mood remains independent of workload changes, though different task difficulties influence both workload and self-reported mood. Additionally, neuroticism affects mood and performance, potentially impacting ATCOs’ work life [37].

Most ATCO-related studies have focused on task complexity and mental workload rather than on investigating stress impacts. However, key neurophysiological findings include enhanced beta EEG band activity, frontal brain activation asymmetry, increased galvanic skin response (GSR), and elevated heart rate (HR) variability [19].

These neurophysiological changes directly relate to glucose regulation through well-established mechanistic pathways. Enhanced beta EEG activity and increased heart rate variability during high-stress events reflect sympathetic nervous system activation, triggering immediate catecholamine release (epinephrine and norepinephrine). This acute stress response promotes rapid hepatic glycogenolysis and gluconeogenesis, potentially causing glucose excursions of 50–100 mg/dL within minutes during emergency scenarios like radar failures or aircraft conflicts. Simultaneously, the elevated GSR indicates cortisol release via HPA axis activation, sustaining hyperglycemia through continued gluconeogenesis and reduced peripheral glucose uptake, effects that may persist for hours after the stressful event resolves.

Selecting appropriate and reliable stress measurements in ATCOs’ environments remains challenging due to the wide spectrum of working conditions, ranging from medium-stress scenarios to high-stress events. Here are some practical examples with varying degrees of stress:

(i) High-complexity area conflict (medium stress): two aircraft may appear on the radar screen while approaching each other, so the ATCOs have to manage vertical and horizontal separation between them with a minimal response and conflict resolution time relative to aircraft speeds in the presence of departing, arriving, and opposite traffic.
(ii) Conflict detection due to transponder Mode C failure (medium stress): Transponder-based altitude (i.e., flight level) information may get lost from the controller’s working position perspective for short periods (i.e., some 20 s). In such conditions, an aircraft might suddenly appear on the radar screen at the same flight level as other aircraft, thus causing a flashing alert from the traffic conflict detection system.
(iii) Social pressure (medium stress): Social pressure and distraction can come from someone close to the ATCO using a mobile phone loudly.
(iv) Radio noise (high stress): Noise from one of an aircraft’s radio systems could suddenly—and hopefully temporarily—make communication with the pilot relatively poor and thus cause difficulty in understanding and providing commands while monitoring and managing the other traffic components.
(v) Emergency descent (high stress): an aircraft can declare an emergency descent, in which case the ATCO has to inform all pilots in close positions and instruct them to take evasive action against sudden crossings and conflicts.
(vi) Lost radar images (high stress): When radar images suddenly, yet temporarily, fade out from the screen, ATCOs have to switch to paper strips only, with lost situational awareness involving aircraft, supporting tools, and safety and efficiency measurements.

Studies simulating these events have demonstrated that high-stress events trigger cognitive and hormonal responses, significantly affecting performance and safety. Importantly, both ATCOs and supervisors tend to underestimate stress levels during actual stress [19].

ATCO stress extends beyond air traffic activity and potential conflicts [38]. All work depletes psychophysical resources, potentially enhancing stress and impacting motivation and job satisfaction [20]. Despite rigorous selection processes including psycho-technical tests, poor stress management can directly cause performance deterioration under conditions involving time pressure, traffic demands, fatigue from circadian shift desynchronization, and equipment limitations [39].

Studies involving pressing radio communications (RC) have shown ATCOs experience significantly higher subjective and objective stress levels, including altered brain rhythms and increased skin conductance, with verbal exchanges showing inverse correlation with communication pressure [19]. Research comparing ATCOs to other high-stress occupations (maritime navigators) reveals that while they generally rate their lives as controllable and predictable, some experience occupational burnout at rates similar to psychiatrists [20].

Early 2000s research identified mental load, time pressure, and responsibility burden as primary ATCO work stress (WS) components, with psychosomatic symptoms more prevalent in regional center radar operators than airport tower controllers, though at lower rates than the general population [21]. Interestingly, daily blood pressure does not systematically increase in ATCOs, suggesting these highly selected and trained individuals maintain composure under pressure [22].

**Figure 1:**
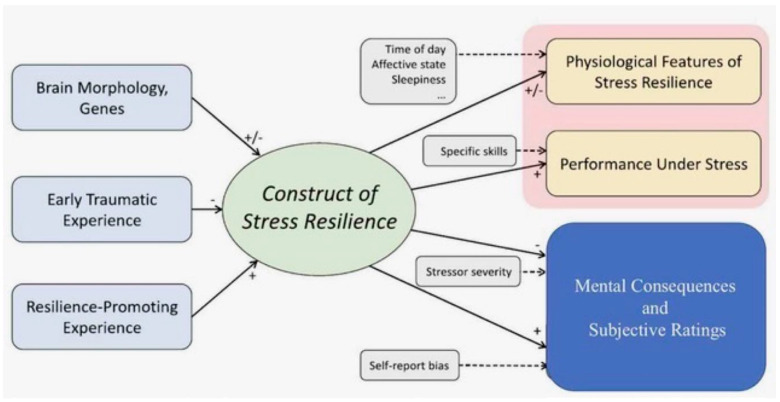
Schematic diagram of stress resilience construct. Left side: most relevant resilience determinants. Right side: most common resilience outcomes. Gray boxes and dashed arrows: disturbance variable positively (+) or negatively (-) influencing outcomes (modified from [40]).

While ATCOs are typically young, intelligent, multitasking, and motivated, not all demonstrate stress-resilience, primarily due to awareness that their performance is critical for human safety. Recent multimodal approaches analyzing various physiological measures (electromyography (EMG), electrodermal activity (EDA), resting heart rate variability (HRV), respiratory sinus arrhythmia (RSA), acoustic startle response (ASR) have been deployed to predict ATCO performance and assess stress-resilience cost-effectively [22,40]. As shown in Figure 1, sophisticated machine learning analysis revealed that when appropriately combined, these individual stress resilience indicators effectively identified candidates’ ability to cope with ATCO-specific task performance requirements under realistic occupational stress conditions. These methods could potentially serve as valuable supplements to traditional selection tests for both immediate job placement and long-term performance evaluation [41].

### 3.2. Stress-Induced Pathophysiological Changes

ATCO stress often stems from the unpredictability of critical situations that disrupt the routine workload. Zeier et al. [23] assessed psycho-physiological stress reactions by sampling saliva before and after working sessions during light and heavy traffic in 158 ATCOs. Unexpectedly, marked increases in salivary immunoglobulin A (sIgA) and cortisol occurred without significant interactions. However, neither absolute values nor autonomic lability scores correlated with actual workload, perceived effort, or session duration. Notably, sIgA values were higher during the light traffic session, while cortisol increased more during heavy traffic. The sIgA response did not correlate with cortisol or perceived workload, while the cortisol response correlated with both workload levels. The authors concluded that sIgA increases reflected positive emotional engagement, suggesting its potential use in differentiating between eustress and distress or successful and unsuccessful coping abilities. Eustress refers to the beneficial or positive form of stress that arises when individuals perceive challenges as opportunities for growth, achievement, or enjoyment, rather than as threats. It is marked by positive emotions, increased motivation, and improved performance or personal development. Unlike distress, which is harmful and overwhelming, eustress helps individuals adapt, learn, and thrive in demanding situations [23].

These cortisol patterns reveal critical mechanistic insights for ATCO glucose regulation. The correlation between cortisol response and workload levels indicates that operational stress intensity directly modulates glucose metabolism through dose-dependent HPA axis activation. In ATCOs with diabetes, this translates to predictable glucose elevations: acute high-traffic scenarios may cause immediate 30–50 mg/dL glucose rises through catecholamine action, while sustained cortisol elevation during prolonged busy periods can maintain glucose levels 20–40 mg/dL above baseline for 2–4 h post-shift. The finding that sIgA response was independent of cortisol suggests that eustress engagement may activate different neuroendocrine pathways that could potentially buffer against stress-induced hyperglycemia, offering a protective mechanism in well-adapted ATCOs [42].

Air Force ATCO studies using simulated TRACON (Terminal Radar Approach Control) scenarios with varying aircraft numbers and traffic complexity have identified significant effects on performance measures, including EEG, eye blink patterns, heart rate, and respiration [43]. Recent research has demonstrated that cognitive workload can be effectively detected through EEG analysis, with mental fatigue significantly impacting cognitive function, response time, and vigilance [24]. The cognitive demands require continuous use of working memory alongside selective attention, focus, speed of perception, activity management, auditory attention, planning, decision-making, and reasoning abilities [25,44]. Additional occupational health concerns in ATCOs include vocal tract discomfort (VTD), which occurs at twice the rate of hoarseness and correlates with perceived environmental stress levels [19,45,46]. Depression studies using the Zung Self-Rating Depression Scale revealed patterns similar to other population groups, with approximately 75% of individuals with symptoms not receiving treatment, suggesting the need to allow many to continue working [26].

Physical inactivity within confined environments presents another challenge, similar to electronic intensive care unit workers who experience weight gain, fatigue, and concentration difficulties from repetitive monitoring activities and prolonged multi-screen computer work [47]. Assessment tools like the Task-load Efficiency Safety-Buffer Triangle (TEST) have been developed to monitor changes in task load, efficiency, and safety buffers, providing insights into controllers’ working styles and supporting supervisor decisions [48].

Brazilian studies of ATCOs revealed that executive brain functioning, including mental flexibility, strategic planning, and inhibitory control, exceeded general population averages regardless of working in approach control or aerodrome tower control. However, ATCOs with ≤5 years seniority displayed better cognitive test scores than more experienced colleagues [49]. Sixteen percent experienced stress with predominant physical symptoms, while 25% of ATCOs experienced excessive daytime sleepiness, particularly those at high-traffic units with 24 h alternating shifts [43].

Auditory processing abilities were assessed using the Synthetic Sentence Identification Test and speech-in-noise test in 30 ATCOs compared to controls. ATCOs demonstrated superior right-ear figure-ground ability despite reporting higher rates of fatigue, burnout, and work-related stress [28,50,51]. Modern research has established that ATCOs possess enhanced cognitive abilities, including time perception, working memory, reasoning, attention, decision-making, and planning, with core functions including inhibitory control, task-switching, visuospatial ability, and breadth of attention [29,30,52,53].

Comparative studies of experienced ATCOs (≥10 years) versus less experienced colleagues and Aeronautical Information Service Operators revealed that highly experienced ATCOs showed significantly lower immune cell activity at shift end, decreased hemoglobin and blood cell counts, and increased morning cortisol concentrations, suggesting that chronic stress significantly affects immune responses [31]. However, these experienced ATCOs demonstrated greater focus, sustained attention, mental manipulation, and resistance to interference, indicating high-level attentional capacity developed over years of experience [54].

### 3.3. Stress Might Affect Workers with Diabetes

Sleep deprivation (SD) represents a common stressor that can arise from sudden job requirements and inappropriate shift schedules. SD typically increases stress hormone concentrations, including cortisol, epinephrine, and norepinephrine, and impairs attention and working memory. However, research indicates that SD does not cause significant changes in glucose and inflammatory marker levels in healthy volunteers [26].

Cognitive aspects play a crucial role in diabetes management under stress. Cognitive load can affect decision-making processes related to insulin dosing and dietary choices. When stressed, cognitive resource depletion may lead to poorer self-management behaviors, such as neglecting blood glucose monitoring or miscalculating carbohydrate intake. Iavicoli et al. [55] recommend education on potential risks, proper organizational interventions, and tight metabolic control assessment for well-compensated, motivated workers with diabetes involved in shift work and high-risk environments.

**Figure 2:**
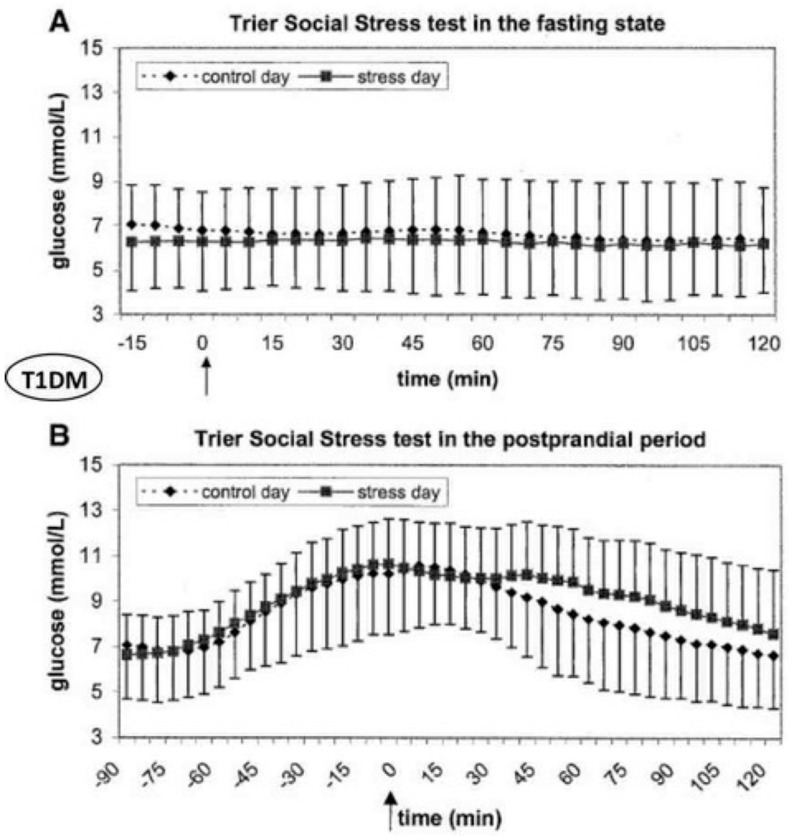
Continuous glucose monitoring in 40 subjects with type 1 diabetes was divided into two equally distributed groups according to basal and postprandial state and analyzed with (stress day) or without (control day) the Trier Social Stress Test (modified from [48]).

Studies using the Trier Social Stress Test (TSST) in people with Type 1 Diabetes Mellitus (T1DM) found no glucose changes in the fasting state but significantly delayed glucose decrease in the postprandial period (see Figure 2) [56].

**Figure 3:**
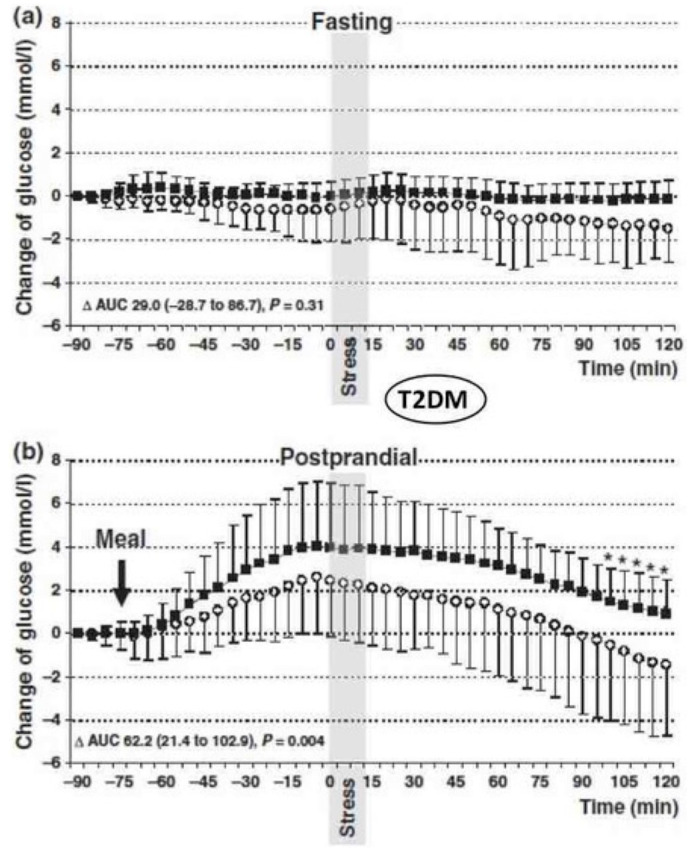
Continuous glucose monitoring levels under fasting and postprandial conditions in people with T2DM; open circles = control day; closed circles = stress day (modified from [49]).

The differential glucose responses between T1DM and T2DM during acute stress testing provide crucial mechanistic insights for ATCO diabetes management. In the operational environment, acute stressors (aircraft emergencies, equipment failures) would cause minimal glucose disturbance in insulin-treated controllers during fasting states. However, if these events occur during meal periods, the delayed glucose clearance could result in prolonged hyperglycemia lasting 1–2 h. In contrast, T2DM patients undergoing similar stress testing showed significantly higher blood pressure, heart rate, salivary cortisol, and blood glucose concentrations compared to non-stress days (*p* = 0.005) (see Figure 3).

This response resulted from patients’ residual insulin response to stimuli, such as increased post-meal glucose concentrations [57]. For ATCOs with T2DM, significant stress-induced glucose elevations suggest that chronic occupational stress may progressively impair insulin sensitivity through sustained cortisol elevation, creating a cumulative metabolic burden distinct from acute stress responses [27,50]. In addition, T2DM has an increased risk of obesity that can exacerbate fatigue and reduce alertness, further compromising performance in high-stress, safety-critical environments like those of ATCOs.

Several studies confirm that psychosocial stress affects insulin resistance through HPA axis activation, making insulin less effective and causing glucose dysregulation, though without dramatic elevations [58,59]. Cognitive appraisal of stressors, how individuals perceive and interpret stress, significantly influences physiological responses and diabetes management. Workers perceiving high-stress situations as challenges may respond differently from those viewing them as threats, affecting emotional responses, coping strategies, and ultimately their glycemic control.

Mathematical models incorporating physical activity and acute psychological stress (APS) measures have been designed to trigger insulin pump adjustments for improved diabetes management. Studies of competitive stress show glucose levels may briefly exceed the 270 mg/dL pilot threshold without food consumption, similar to high-intensity athletic competition effects in T1DM athletes [60,61].

Research examining continuous glucose monitoring (CGM)-based glucose concentrations in T1DM patients waking to 4 a.m. alarms found clinically non-relevant 18 ± 6 mg/dL glucose increases, with even smaller changes when nurse assistance was provided [62]. Studies confirm that people with diabetes under high work-stress conditions experience slightly higher HbA1c levels, with higher perceived stress risk associated with elevated HbA1c [63].

Importantly, research evaluating daily life glucose changes in pump-wearing T1DM patients found a significant correlation between stress and the percentage of hypoglycemic events. This unexpected finding, despite high stress hormone output, resulted from high perceived psychological stress, leading participants to reduce carbohydrate intake without corresponding insulin reduction. This highlights the need for proper stress management education, including accurate insulin dose adaptation to changed carbohydrate intake during psychosocial stress [64].

### 3.4. Diabetes Management Considerations Within a Complex Stress Environment

The interaction between occupational stress, shift work, and glycemic control in ATCOs with diabetes involves multiple confounding variables that must be carefully considered for medical certification.

High traffic volume, fear of causing accidents, equipment limitations, and the need for constant vigilance requirements are major workplace stressors in ATCOs [64]. These acute stressors can trigger rapid glucose elevations through sympathetic nervous system activation, while chronic occupational stress may cause sustained hyperglycemia via cortisol-mediated pathways. Our shift-pattern analysis reveals that night-shift work compounds these effects through circadian rhythm disruption and irregular meal timing.

Research demonstrates that cortisol-induced stress is associated with various socioeconomic determinants, including monthly income, job stability, household responsibilities, and marital stress [65,66].

The relatively high compensation and job security typically associated with ATCO positions may serve as protective factors against some forms of psychosocial stress commonly observed in other populations. However, the unique demands of shift work, particularly the alternation between day and night shifts identified in our review, may exacerbate stress responses through disruption of circadian rhythms and family life balance. Our review demonstrates that acute stress affects glucose regulation differently in Type 1 versus Type 2 diabetes, with important practical implications for ATCOs. Shift work compounds stress-induced glucose dysregulation through circadian rhythm disruption, irregular meal timing, and sleep deprivation. However, the high motivation for glucose control among aviation professionals, combined with modern continuous glucose monitoring technology, may mitigate some of these risks. The individualized assessment approach we propose must therefore consider not only diabetes management competency but also the specific stressors and shift patterns each ATCO faces.

The mechanistic understanding of stress-glucose interactions in ATCOs requires individualized management strategies that account for both acute and chronic stress patterns. Continuous glucose monitoring data should be analyzed in relation to specific operational stressors: rapid glucose spikes (>50 mg/dL in <30 min) likely reflect acute sympathetic activation during high-stress events, while sustained elevations (>20 mg/dL above baseline for >2 h) suggest cortisol-mediated effects requiring different therapeutic approaches. Experienced ATCOs demonstrate stress resilience over time through adaptive downregulation of stress hormone responses, potentially explaining why experienced controllers show different cortisol patterns. This adaptation could provide a physiological basis for allowing continued certification in insulin-treated ATCOs who demonstrate stable glucose control during documented high-stress operational periods.

## 4. Novelty, Strengths, and Limitations

### 4.1. Novelty and Strengths

The core innovation of this narrative review lies in advocating for individualized, case-by-case assessment of insulin-treated ATCOs rather than blanket exclusions. This represents a significant departure from traditional aviation medical certification approaches that typically apply categorical restrictions to insulin-dependent diabetes. Several novel contributions distinguish this work: first, this review uniquely incorporates up-to-date diabetes management technology (advanced hybrid pumps, continuous glucose monitoring) into the aviation safety discussion. This technological perspective has not been comprehensively addressed in previous aviation medical literature. Second, the behavioral psychology element we introduced, i.e., that aviation professionals with diabetes are likely to maintain tighter glucose control due to career stakes, is novel in this regulatory context. Third, emphasizing the societal investment in ATCOs’ training as justification for accommodation represents a resource-based ethical argument that is underexplored in aviation medical policy. Rather than simply arguing restrictions, this review proposes a specific framework for rigorous and structured evaluation, offering a constructive approach that differs from purely oppositional arguments in existing literature.

### 4.2. Limitation

This review has several methodological limitations that must be acknowledged. First, the search strategy was limited to PubMed, potentially excluding relevant articles indexed in other databases. While most medical articles are indexed on PubMed, this limitation may have resulted in incomplete coverage of available literature. Second, high-quality articles in languages other than English were excluded (we included just two papers in Portuguese and in Spanish), thus potentially missing important perspectives from international aviation medical research. Third, the absence of formal systematic review methods increases the risk of bias and makes replication difficult due to the lack of standardized procedures. Fourth, significant methodological heterogeneity exists across the included studies, particularly regarding the control of confounding variables.

The interaction between shift patterns and physiological stress responses represents a critical confounding variable that was inconsistently controlled across studies. Our review identified that 84.6% of ATCOs experiencing excessive daytime sleepiness were based at units operating 24 h alternating shifts, yet many studies failed to adequately stratify results by shift patterns [67]. Additionally, individual differences in stress resilience, years of experience, and coping mechanisms may have obscured or exaggerated reported associations between occupational stressors and physiological outcomes. The finding that ATCOs with ≥10 years of experience showed different cortisol patterns and immune responses compared to less experienced controllers suggests that adaptation mechanisms develop over time. This temporal factor potentially confounds cross-sectional comparisons and highlights the need for a longitudinal study design. Finally, there is minimal synthesis of conflicting evidence, especially regarding the variability of stress reactivity among ATCOs and people with diabetes. This gap underscores the need for research that explicitly addresses these differences and provides a more nuanced understanding of stress impacts across different populations. These methodological limitations highlight the need for more standardized approaches to studying ATCO stress that systematically accounts for shift patterns, experience levels, and individual resilience factors in future research.

## 5. Future Research Directions

To address the limitations identified in current ATCO stress research, several key research priorities should be pursued: First, future studies should implement comprehensive stress assessment models that incorporate both occupational and socioeconomic stressors, including validated measures of financial security, family responsibilities, and social support systems. Second, researchers should standardize shift pattern documentation and stratify analyses by day/night work schedules to better understand circadian-related confounding effects. Longitudinal study designs are critically needed to track stress responses and adaptation mechanisms over career progression, accounting for the protective effects of experience while monitoring for burnout patterns. These studies should specifically focus on glucose regulation outcomes in ATCOs with diabetes, examining how stress-glucose interactions evolve with professional experience and technological advances in diabetes management. Additionally, future research should develop multivariate models that can parse the relative contributions of occupational demands, shift patterns, socioeconomic factors, and individual resilience characteristics to overall stress burden and their specific impacts on glucose control. Finally, prospective studies are needed to validate the proposed individualized assessment framework for insulin-treated ATCOs, establishing evidence-based criteria for medical certification that balance aviation safety with occupational equity. These validation studies should incorporate real-world continuous glucose monitoring data during documented operational stressors to establish evidence-based thresholds for safe aviation practice. This research agenda would provide the empirical foundation necessary for developing evidence-based policies that move beyond categorical exclusions toward individualized risk assessment in aviation medical certification.

## 6. Conclusions

Managing insulin-treated diabetes requires significant resources and presents daily challenges. However, professionals with diabetes are acutely aware of the high risk they face, including both life-threatening complications and the potential loss of employment and related income. Once metabolic control is achieved, these professionals are highly motivated to maintain glucose stability and avoid extreme excursions that could compromise their certification. Present diabetes management technology, particularly advanced hybrid pumps that enable semi-automatic glucose control throughout the day, significantly enhances the ability of insulin-treated ATCOs to maintain optimal glucose ranges. With consistent commitment to glucose management, these professionals are likely to achieve superior metabolic control compared to the general diabetes population. Given adequate glucose control maintained over a sufficient period during working hours, preventing qualified professionals with insulin-treated diabetes from receiving ATCO certification appears no longer appropriate, and it is necessary to reformulate the normative. A structured pathway to fitness certification represents an approach to consider for aviation medical policy. Aeromedical examiners should employ a rigorous, individualized evaluation process when assessing insulin-treated ATCOs for certification. This framework should include the following operational safeguards: (i) continuous glucose monitoring systems with real-time alerts and threshold protocols; (ii) standardized stress monitoring protocols during duty periods; (iii) periodic high-fidelity simulator evaluations under various operational scenarios to assess glucose stability under controlled stress conditions; (iv) mandatory backup glucose management systems and predefined fitness-for-duty protocols; and (v) regular peer review of glucose management performance during operational shifts. This individualized approach offers multiple benefits: increased professional motivation, enhanced job satisfaction, and optimal utilization of society’s substantial investment in ATCO training, particularly valuable when experienced controllers develop diabetes after qualification.

### Future Research Imperatives

Empirical research is critically needed to evaluate the real-world safety profile of insulin-treated ATCOs. Priority studies should assess the effectiveness of proposed safeguards through longitudinal research incorporating operational stress testing, glucose variability analysis across different shift patterns, and the systematic evaluation of decision-making capacity under various glycemic conditions. Generating robust, context-specific evidence will be essential for informing future regulatory decisions that balance operational safety with professional inclusion in this highly specialized field. The ultimate goal is evidence-based policy that moves beyond categorical exclusions toward individualized risk assessment, ensuring both aviation safety and the equitable treatment of qualified professionals with diabetes.

## Figures and Tables

**Table 1:** Selected Articles on ATCO workload and stress management.

Authors	Participants	Methods	Main Results
Borghini, G. et al. (2020) [19]	ATCOs (*n* = 16)	A simulation of air traffic management collected subjective data (stress perception) and neurophysiological data (brain activity, heart rate, galvanic skin response).	EEG, ECG, and GSR combinations create a stress index to measure stress variations during ATM activities.
Makara-Studzińska, M. et al. (2020) [20]	Maritime navigators (*n* = 54); ATCOs (*n* = 88)	Set of questionnaires for occupational burnout, perceived stress, and seniority.	Workplace demands and employee predispositions impact occupational burnout, life evaluation, and personal resources.
Costa, G. (2000) [21]	ATCOs aged 23–59 years (*n* = 762)	Clinical evalutaion and a set of questionnaires	Work stress appeared related mainly to mental charge, aggravated by time pressure and high responsibility.
Sega, R. et al. (1998) [22]	ATCOs (*n* = 80); Controls (*n* = 216)	Clinical evaluations	ATCOs adequately cope with the stress inherent to the job.
Zeier, H. et al. (1996) [23]	ATCOs (*n* = 199)	Before and after two working sessions at a radar workplace, 2 min samples of whole unstimulated saliva were collected for about 100 min.	The demanding work of ATCs increases salivary cortisol and, sIgA.
Hui, L. et al. (2024) [24]	ATCOs (*n* = 41)	A cognitive workload detection method for air traffic controllers based on mRMR and fewer EEG channels.	Employing EEG equipment for the detection of the cognitive workload
Balta, E. et al. (2024) [25]	ATCOs (*n* = 12)	The communication device, and the digital recorder of the control tower plus blood and skin conductunce measures	Mental workload modulates time perception in complex, real-world environments.
Barrett, J. et al. (1978) [26]	ATCOs aged 25–49 years (*n* = 416)	3-year perspectives study of health change	Two patterns of depression were noted: one acute and episodic, resembling “episodic minor depressive disorder,” and another chronic and fluctuating, akin to “chronic and intermittent minor depressive disorder,” with significant symptoms for over half the year.
Yu, X. et al. (2025) [27]	ATCOs (*n* = 24)	Simulator known as Endless ATC is used to simulate basic ATC operations on a radar map display	The semi-supervised learning approach can accurately provide continuous workload estimations for ATCOs and outperforms the baseline models.
Villar ACNWB et al. (2019) [28]	ATCOs (*n* = 30); Military personnel and civilians (*n* = 39)	Attention, communication, and health questions, plus speech-in-noise and SSI monotic tests for closure and figure-ground assessment.	ATCOS reported higher fatigue and stress but outperformed the control group in figure-ground and closure tasks. There was a 5.59 times greater likelihood of being CTA for stress and a 1.24 times greater likelihood of identifying phrases in right-ear monotic listening.
Bernhardt et al. (2019) [29]	ATCOs (*n* = 47: 27 experienced; 20 less experienced)	Clinical trial on inhaler techniques	The EEG engagement metric varied by experience, with less experienced controllers more engaged than experienced ones. The EEG workload metric and pupil diameter were influenced by workload changes but did not distinguish between experience levels.
Borghini et al. (2017) [30]	ATCOs Expert (*n* = 15) ATCOs Students (*n* = 22)	Air Traffic Management Simulation	Brain features may identify and differentiate Skill and Rule Knowledge levels, allowing for objective assessment of cognitive control behaviors in real-life situations.
Ribas, V.R. et al. (2011) [31]	ATCOs (*n* = 30; ATCo > 10 − *n* = 15; TCo < 10 − *n* = 15); Aeronautical Information Service Operators (AIS: *n* = 15; AIS > 10 *n* = 8; AIS < 10 *n* = 7)	Blood samples were drawn at 8:00 a.m. and 2:00 p.m.	The ATCo ≥ 10 group had a lower monocyte phagocytosis rate at 2:00 p.m. compared to 8:00 a.m. They also had reduced hemoglobin, mean corpuscular hemoglobin concentration, platelets, and leukocytes, with higher cortisol at 8:00 a.m. Compared to other groups, they exhibited lower levels of phagocytosis, hemoglobin, platelets, leukocytes, basophils, and nitric oxide at 2:00 p.m.

## Data Availability

No new data were created.

## Abbreviations

The following abbreviations are used in this manuscript:

ATCOs	air traffic controllers
PwD	people with diabetes
TIR	time in range
LADA	latent autoimmune diabetes in adulthood
TVF	traditional vigilance format
SART	Sustained Attention to Response Task
XIM	eXperience Induction Machine
EEG	electroencephalogram
GSR	galvanic skin response
HR	heart rate
RC	radio communications
MNs	maritime navigators
WS	work stress
SR	stress resilience
EMG	electromyography
EDA	electrodermal activity
HRV	heart rate variability
RSA	respiratory sinus arrhythmia
ASR	acoustic startle response
sIgA	salivary immunoglobulin A
TRACON	Terminal Radar Approach Control
NASA TLX	NASA Task Load Index
VTD	vocal tract discomfort
eICU	electronic intensive care unit
eNurses	electronic nurses
ePhysicians	electronic physicians
TEST	Task-load Efficiency Safety-Buffer Triangle
ATC	Aerodrome Tower Control
APC	Approach Control
WCST	Wisconsin Card Sorting Test
APPCC	Approach Control Center (APPCC)
SSI-ICM	Synthetic Sentence Identification Test–Ipsilateral Competitive Message
SIN	speech-in-noise test
PASAT	Paced Auditory Serial Addition Test
SD	sleep deprivation
TSST	Trier Social Stress
T1DM	Type 1 Diabetes Mellitus
T2DM	Type 2 diabetes mellitus
HPA	hypothalamic–pituitary–adrenal
APS	acute psychological stress
STAI	State-Trait Anxiety Inventory
ICII	Insulin Confidence Index
CGM	continuous glucose monitoring
